# Overcoming tumor immune evasion with an unique arbovirus

**DOI:** 10.1186/s12967-014-0349-0

**Published:** 2015-01-16

**Authors:** Bruce Lyday, Tony Chen, Santosh Kesari, Boris Minev

**Affiliations:** PrimeVax, LLC, Garden Grove, USA; Department of Neurosciences, Translational Neuro-Oncology Laboratories, UC San Diego, La Jolla, CA 92093 USA; Moores UCSD Cancer Center, UC San Diego, La Jolla, CA 92093 USA; Division of Neurosurgery, UC San Diego, La Jolla, CA 92093 USA; Genelux Corporation, San Diego Science Center, San Diego, CA 92109 USA

**Keywords:** Death Receptors, Dendritic cell, Dengue fever, Hyperthermia, Immunotherapy, Immune Evasion, Spontaneous Regression, Soluble TRAIL

## Abstract

Combining dendritic cell vaccination with the adjuvant effect of a strain of dengue virus may be a way to overcome known tumor immune evasion mechanisms. Dengue is unique among viruses as primary infections carry lower mortality than the common cold, but secondary infections carry significant risk of hypovolemic shock. While current immuno-therapies rely on a single axis of attack, this approach combines physiological (hyperthermic reduction of tumor perfusion), immunological (activation of effector cells of the adaptive and innate immune system), and apoptosis-inducing pathways (sTRAIL) to destroy tumor cells. The premise of using multiple mechanisms of action in synergy with a decline in the ability of the tumor cells to employ resistance methods suggests the potential of this combination approach in cancer immunotherapy.

## Introduction

Combining dendritic cell vaccination with the adjuvant effect of a strain of dengue virus may be a way to overcome known tumor immune evasion mechanisms. Dengue is unique among viruses as primary infections carry lower mortality than the common cold, but secondary infections carry significant risk of hypovolemic shock [1,2]. While current immuno-therapies rely on a single axis of attack, this approach combines physiological (hyperthermic reduction of tumor perfusion), immunological (activation of effector cells of the adaptive and innate immune system), and apoptosis-inducing pathways (sTRAIL) to destroy tumor cells. The premise of using multiple mechanisms of action in synergy with a decline in the ability of the tumor cells to employ resistance methods suggests the potential of this combination approach in cancer immunotherapy.

Recently, three new classes of tumor immunotherapeutic agents have achieved clinical and regulatory success. Amgen’s talimogene laherparepvec (OncoVex^GM-CSF^), a recombinant oncolytic virus, has demonstrated clinical responses and a favorable safety profile in advanced melanoma, a challenging indication with significant unmet needs [3]. Dendreon’s sipuleucel-T (Provenge™) for prostate cancer, and Bristol-Meyers Squibb’s (BMS) ipilimumab (Yervoy™) won first-in-class FDA approval for cancer vaccines and immune checkpoint blockade agents. The very recent FDA approval of Keytruda, a PD-1 blocking agent, was a promising addition to the immuno-oncology targeting approaches [http://www.fda.gov/NewsEvents/Newsroom/PressAnnouncements/ucm412802.htm]. However, the high cost of these agents, combined with high toxicity for Yervoy™ [4], and modest survival benefit for Provenge™, have dampened the initial enthusiasm [5,6]. Recently, BMS’s combination of a CTL-4A blocking agent (Ipilimumab) [7] and a PD-1 blocking agent (Nivolumab) [8] demonstrated superior response rates [3]. However, although the spectrum of adverse events observed among patients treated with the concurrent regimen was qualitatively similar to previous experience with nivolumab or ipilimumab monotherapy, the rate of adverse events was higher among patients treated with the combination therapy. These, and other studies, have proven that dendritic cells (DC) can induce Ag-specific cytotoxic T lymphocytes (CTL), and that checkpoint inhibitors can break T-cell tolerance to self-peptides. Another new immunotherapy strategy involves creating chimeric antigen receptor (CAR) T-cells as an adoptive cell therapy. These genetically modified T-cells have high affinity for tumor antigens, and recent data points to their efficacy against some hematologic malignancies [9]. However, the solid tumors employing numerous immune evasion mechanisms described below appear much more resistant to CAR T-cell therapy.

All of the above technologies share a common weakness in that they are attacking tumor cells along a single axis of mechanism of action. They have shown that early incomplete clinical responses to single-mechanism drugs are often followed by relapse, due to the emergence of resistant tumor clones [10]. These new technologies rely primarily on CTL, using specific T-Cell receptors to identify and kill tumor cells. Over time, resistant tumor clones with low or absent major histocompatibility complex (MHC) expression often emerge under selective pressure by CTL [11]. High mutation rates allow tumor cells to turn genes on and off at random, employing multiple evasion mechanisms, thereby allowing them to evade the CTL induced by modern immunotherapies [12]. While researchers have identified the genotypic and phenotypic profiles of CTL that can generate an effective response in the patient, durable, complete responses remain rare [13]. Reviewing cases of spontaneous regression in the context of extended febrile infections may hold critical clues for researchers in cancer immunotherapy [14].

An ideal immunotherapeutic strategy would employ multiple mechanisms to overcome all of the tumor immune evasion mechanisms and resistance factors seen in advanced tumors.

### Spontaneous regression of cancer and the febrile effect

**Figure 1:**
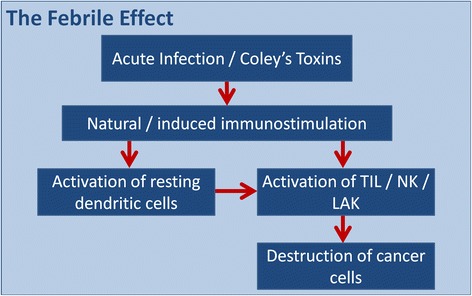
**Sequence of events showing how immune responses to pathogens can impact the immune response to tumor cells.**

Spontaneous regression (SR) is a rare event in cancer [14]. A common factor in many cases is a systemic infection leading to a prolonged high fever in excess of 39.5°C. While melanomas and sarcomas are the two indications representing the most SR, regressions are also observed in breast, lung, and other tumor types. In the 1880’s, Dr. William Coley noticed that cancer patients with post-surgical infections, usually with *Erysipelas* bacteria, had improved outcomes over those who did not have such infections [15]. His observations lead to preparation of Coley’s Toxins, suspensions of Gram^+^ bacterial cell extracts, which caused high fever along with immune activation. These mechanisms of action could induce potent immune responses, resulting in tumor regression, as outlined in Figure 1 [16].

Tumors, by virtue of their high mitotic and cellular metabolic rates, are often oxygen deficient [17]. This oxygen deficiency leads to higher utilization of anaerobic pathways to generate adenosine triphosphate (ATP), with the result of higher levels of lactate, and lower pH within the cytoplasm and nucleus [18]. This effect is seen as a gradient, with low-perfusion melanomas and sarcomas most vulnerable, and oxygen-saturated lung and CNS tumors most resistant to the effects of febrile hypo-perfusion [18]. In addition, the tumor perfusion is lower under febrile conditions, leading to a selective peripheral blood network restriction to conserve oxygen to the brain [19]. In doing so, the oxygen supply to the tumor sites is reduced_._ Therefore, by targeting and eradicating these low-perfusion tumor sites with high genetic plasticity, it might be possible to achieve an enhanced response to therapy [20].

The effect of these restrictions on the hypo-perfused tumor sites is an increase of their already high rates of glycolysis [18]. However, perfusion is restricted for both afferent and efferent vessels toward and away from the tumor, so lactate and other waste products accumulate, leading to necrosis [18]. This effect is further compounded by high levels of TNFα, released by the immune system during acute infections [21]. TNFα is an inflammatory cytokine with pleiotropic effects, including direct killing of tumor cells via TRAIL (TNFα-Apoptosis-Inducing-Ligand) [21,22]. A major function of TNFα is to bind with Platelet-Erythrocyte-Cellular-Adhesion Molecule-1 (PECAM-1), opening gap junctions in High Endotheliel Venules (HEV) [21]. Consequently, activated lymphocytes adhere to, and squeeze through the gap junctions in the openings of HEVs, allowing them to engage pathogens and tumor cell targets [23]. While TNFα is an important cytokine in immunotherapy, high levels of TNFα can result in capillary leak syndrome, which, if unchecked, can lead to hypovolemic shock [1,24].

DNA microarray analyses have revealed that hundreds of genetically distinct tumor clones may exist in a single patient with advanced tumor [25]. There is a pattern of negative correlation between O_2_ supply and genetic mutation rates [20]. The majority of agents such as cytotoxic drugs, antibodies, and small molecules, are nearly always blood-borne, exerting a Darwinian selective pressure to tumor clones that evade therapeutic mechanisms [17]. Clones with the lowest perfusion rates have both low drug exposure and high capacity to evade immune system detection, making them resistant to conventional therapies [17,20]. Fever hyperthermia takes advantage of this situation, starving low-flow, resistant clones with mutated phenotypes, leaving more genetically stable clones for elimination by activated lymphocytes and other arms of the immune system [26,27]. Combining fever with activation of CTL and lymphokine-activated killer cells (LAK) could lead to higher response rates [23].

As intracellular pathogens, viruses activate LAK and CTL via a Type I gene signature pathway, e.g., IL-1b, IL-2, IL-7, IL-12, IL-15, IFNα, IFNβ, IFNγ, and GM-CSF [28]. This activity mimics the signature found in responders to cancer immunotherapy [27]. While numerous approaches using oncolytic and recombinant viruses as vectors for tumor antigens have been attempted, the clinical results have been disappointing [29-32]. In light of the progress and limitations made in these areas, the dengue virus is presented as a potential alternative to other virus-based therapeutic approaches.

### Dengue virus as an adjuvant to immunotherapy

Dengue viruses (DV) are small (40 nm) + strand RNA viruses of the *Togaviridae* family, subfamily *flaviviridae*, Group B [33]. DV is unique in that secondary infections carry significant risk of hypovolemic shock, while the primary infections have lower death rates than the common cold virus [2,34].

### Natural course of dengue infections

**Figure 2:**
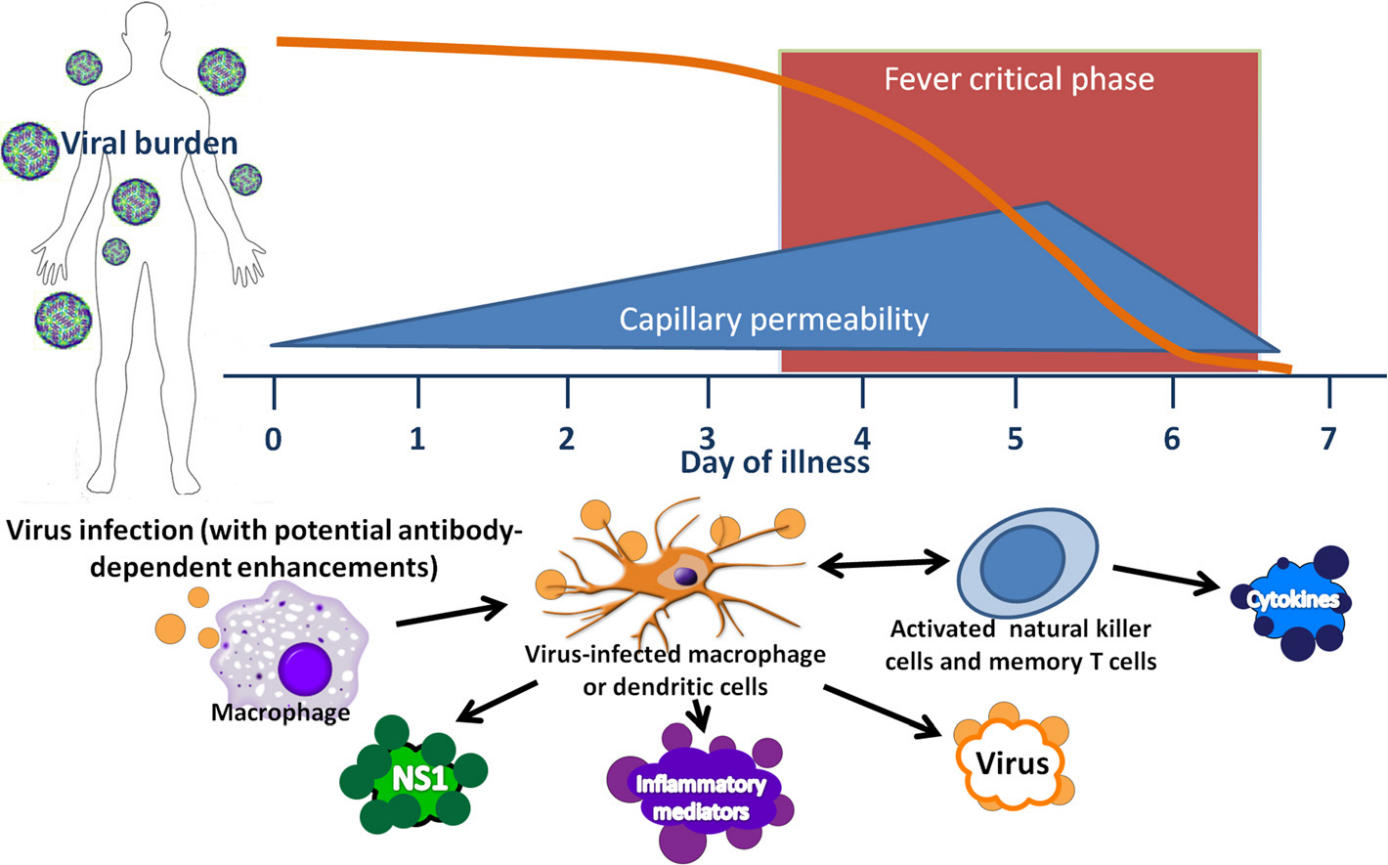
**Dengue fever and mechanisms of the immune response to the virus.** NS1: nonstructural protein 1.

Dengue Fever is an acute self-limiting febrile disease with significant toxicities that are transient and self-limiting in nature in >95% of primary infections [1]. The mechanisms of the immune response to the Dengue virus and fever generation are shown in Figure 2.

**Figure 3:**
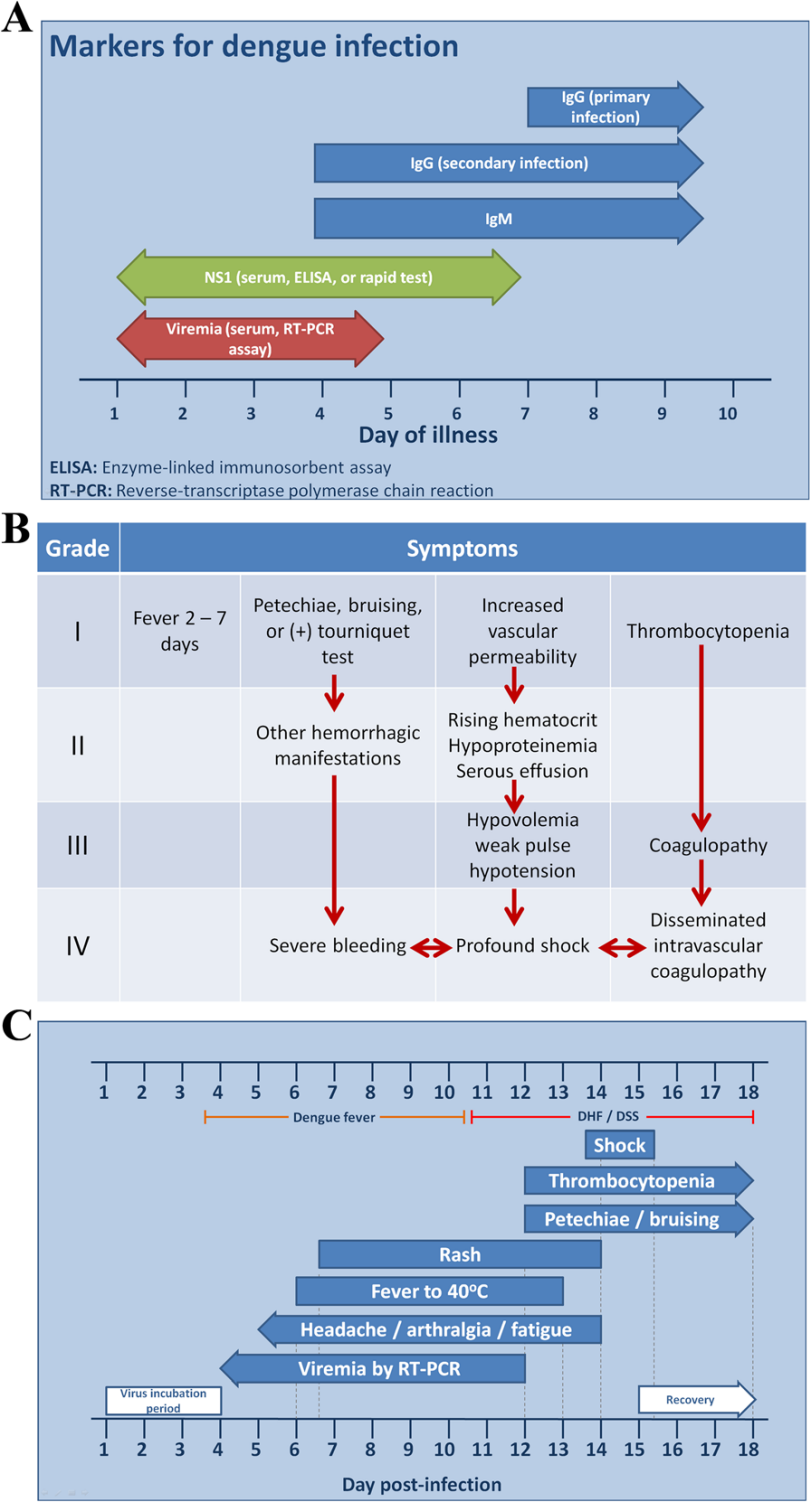
**Course of Dengue Fever, Markers, Grading, and Symptoms. (A)** Laboratory diagnostic options in a patient with suspected dengue infection, **(B)** Progression from Dengue Fever to Dengue hemorrhagic fever (DHF) and **(C)** Timeline and course of clinical signs and symptoms of Dengue.

The virus infects white blood cells of monocyte/macrophage/dendritic cell lineage [35,36] (Figure 2). The infected cells produce interferons, interleukins, and other factors which mediate both specific and non-specific immune responses, including high fever, capillary permeability, rash, joint pain, and elevations of hepatic transferase enzymes [1,22,35,37] (Table 1 and Figure 3).

**Table 1:** **Cytokine levels induced by dengue virus**

**Cytokine**	**Control**	**Post-Adjuvant**	**Increase**	**Data type/Ref #**
IL-1β	9.4 pg/ml	745 pg/ml	79X	In vitro [35]
IL-2	2.1 U/ml	60.3 U/ml	29X	In vivo [37]
IL-7	18 pg/ml	75 pg/ml	4.6X	In vivo [38]
IL-12	Undetectable	270 pg/ml	270X	In vivo [39]
IL-15	5.2 pg/ml	12-31 pg/ml	2-6X	In vivo [40]
IFNα	<6 U/ml	1600 U/ml	267X	In vivo [41]
IFNγ	<0.025 U/ml	0.95 U/ml	16X	In vivo [37]
TNFα	3 pg/ml	210 pg/ml	70X	In vivo [22]
TNFβ	Undetectable	To 605 pg/ml	600X	In vivo [41]
GM-CSF	0.2 pg/ml	10.5 pg/ml	53X	In vitro [42]

A unique feature of DV is that primary infections result in activation of a T_H_1-type response of CD4^+^ and CD8^+^ helper-inducer and cytotoxic-effector CTL, which is also an effective phenotype for tumor immunotherapy [43,44]. Secondary infections activate a T_H_2-type response, with corresponding higher levels of pro-inflammatory cytokines [45,46]. Secondary infections carry a higher risk of complications, including progression to Dengue Hemorrhagic Fever (DHF) [38,45]. The proposed causes of the cytokine shift to T_H_2-type are many, but a major factor appears to be the existence of semi-neutralizing IgG directed against the E glycoprotein.

By binding to, but not fixing, complement for virion destruction, these immune complexes are scavenged by activated Macrophages with up-regulated F^C^ receptors [38]. This increases infection kinetics, leading to higher levels of TNFα and IL-6, with a subsequent increase in capillary permeability pre-disposing to hypovolemic shock, especially in protein-malnourished children and infants [1,46]. This phenomenon is termed the “Immune Enhancement Theory.”

In addition to the above, there are other cases of DHF/DSS, which occur in primary infections that the Immune Enhancement Theory cannot explain. In some of these cases, genetic influences on children with certain HLA haplotypes can lead them to be more susceptible to DHF/DSS [24]. In other cases, many children from low-income areas of Asia, Africa, and Central and South America, lack protein that lead to low serum albumin levels. This is often compounded by a lower Body Mass Index, which provides a higher ratio of vessel surface area to body mass. These children represent a set of physiological risk factors for increased capillary permeability predisposing to shock syndrome upon infection with dengue [1]. Additionally, many of the parents administer aspirin or NSAID drugs to the children, aggravating their plasma leakage.

In conclusion, the chances of DHF/DSS occurring during a primary infection of a well-nourished adult in a clinical setting are very low. In a managed setting, most cases of DHF/DSS can be resolved by prompt administration of replacement fluids [47].

Detection of viral nucleic acid, nonstructural protein 1 (NS1), or IgM sero-conversion is a confirmatory finding in patients with suspected DV infection (Figure 3).

### Vaccine development and safety

A DV epidemic in Brazil had no reported deaths out of 17,440 confirmed cases [48]. A study of elderly Taiwanese found that patients with various pre-existing cancers had a reduced risk of severe DV infection, with an ORR of 0.9 [49]. Pre-existing organ failure, however, doubled the mortality risk [49,50]. Therefore, the proper and safe use of dengue as an immunotherapy agent is predicated on proper fluid balance [1,47].

Disease severity progression from uncomplicated dengue fever (grade 1) to dengue shock syndrome (grade 4) (Figure 3b):

- Dengue fever (DF)
  Febrile illness with 2 or more of the following:
  i. Headache
  ii. Retro-orbital pain
  iii. Myalgia
  iv. Arthralgia
  v. Rash
  vi. Leukopenia
  vii. Hemorrhagic manifestations
  viii. Virus recovery
  ix. Serological response
  x. Temporal occurrence with other cases
- Progression from Dengue Fever to Dengue hemorrhagic fever (DHF)
- Dengue Hemorrhagic Fever (DHF)
  i. DHF has all of the symptoms of Dengue Fever plus:
  ii. Rising hematocrit
  iii. Rise in AST/ALT >3.5 ULN
  iv. Other hemorrhagic signs.
- Dengue Shock Syndrome (DSS)
  i. Rapid / weak pulse, and narrow pulse pressure; or
  ii. Manifestations of hypotension and cold, clammy skin and restlessness
  iii. DSS is treated with fluid replacement and supportive care
- Timeline and course of clinical signs and symptoms of DV infection (Figure 3c)

### Dengue and immunosuppression of cancer patients

The immune suppression seen in patients with advanced cancer is a complex and dynamic process. It involves tolerance to the tumor antigens themselves, which are usually recognized as “self” by CTL [51]. Breaking this tolerance can be achieved by high levels of T_H_1 cytokines, which dengue infection induces [37,51]. An important distinction with DV is that the majority of toxicities are immune-mediated [52]. Children, who develop a more immune-competent systemic response, generally experience a more severe course of disease than adults [1,46]. An article on the dengue epidemic in Taiwan in 2002 listed only 1 serious adverse event (renal failure) among 26 elderly cancer patients with severe dengue [49]. The authors concluded that cancer co-morbidity lowered risk for severe dengue (ORR of 0.9), but pre-existing organ failure doubled the mortality risk. Proper patient selection and strict clinical monitoring of vital signs, especially fluid and electrolyte balance, should mitigate this risk [47].

### The promise of DV as an immunostimulant

DV has many characteristics supporting its use as a potent immune-stimulant in cancer immunotherapy. DV has affinity for immature B-lymphocytes and antigen-presenting cells (APC) of monocyte/macrophage and DC lineage [42]. The kinetics of dengue infection proceed in a linear fashion: injection of >10^6^ pfu/ml by *Aedes* mosquito bite, then, a 4-5 day incubation, followed by a 5-day syndrome characterized by sudden onset of high (to 40.5^°^C) fever, myalgia, arthralgia, photophobia, and rash, followed by complete recovery in the majority of cases [1]. By infecting, but not killing, the APC, DV up-regulates their CD80 and CD83 expression, resulting in a pro-inflammatory T_H_1 cytokine profile [53]. Primary DV infections induce a T_H_1 type response with activated CD4^+^ and CD8^+^ effector T cells as well as LAK cells [37,40,54]. This type of response is seen in patients having complete responses to cancer immunotherapies (Table 2) [25,55].

**Table 2:** **Tumor immune evasion mechanisms and DV infection**

**Immune evasion**	**Dengue counter-attack**
Low levels of MHC on tumor cell prevent CTL recognition [11]	Hi Interferon-γ raises MHC levels by up-regulating MHC gene expression [37,52]
Point mutations in Tumor Peptides prevent TCR binding [12]	LAK/CIK cells target “escaped” tumor cells expressing aberrant peptides or MHC [40,56]
Tumor vessels lack factors for CTL attachment and trafficking [12]	Hi [TNF-a] restores gaps by altering PECAM-1, restores ICAM-1/VCAM-1 expression and P and E-selectins [22,57]
FasL can kill Fas^+^ CTL by triggering apoptosis [12]	Hi [IL-6, 15] protects Fas^+^ CTL by up-regulating FLIP ligand [58,59]
HLA-G protects from NK Cells [12]	Hi [IL-2,7,12,15 raise activation of NK [56,60]
Stromal barriers inhibit CTL [12]	Hi [IFN-γ] activates Macrophages to M_1_ [36]
Myeloid-Derived Suppressor Cells, (MDSC) [61]	iNKT Cells can decrease MDSC [61]
CTL inactivated by TGF-β [12]	T_H_1 cytokines reactivate tolerant CTL [51,62]
Tumor PI-9 blocks CTL killing ([63]	Hi [CD8] & ICAM-1 expression can restore low-avidity CTL recognition and lysis by stabilizing weak interactions between TCR and MHC + self-peptide [64]
T-regulatory cells block CTL [61]	Hi CD4^Helper^ cells overcome CD4^Reg^ cells [37,61]

**Figure 4:**
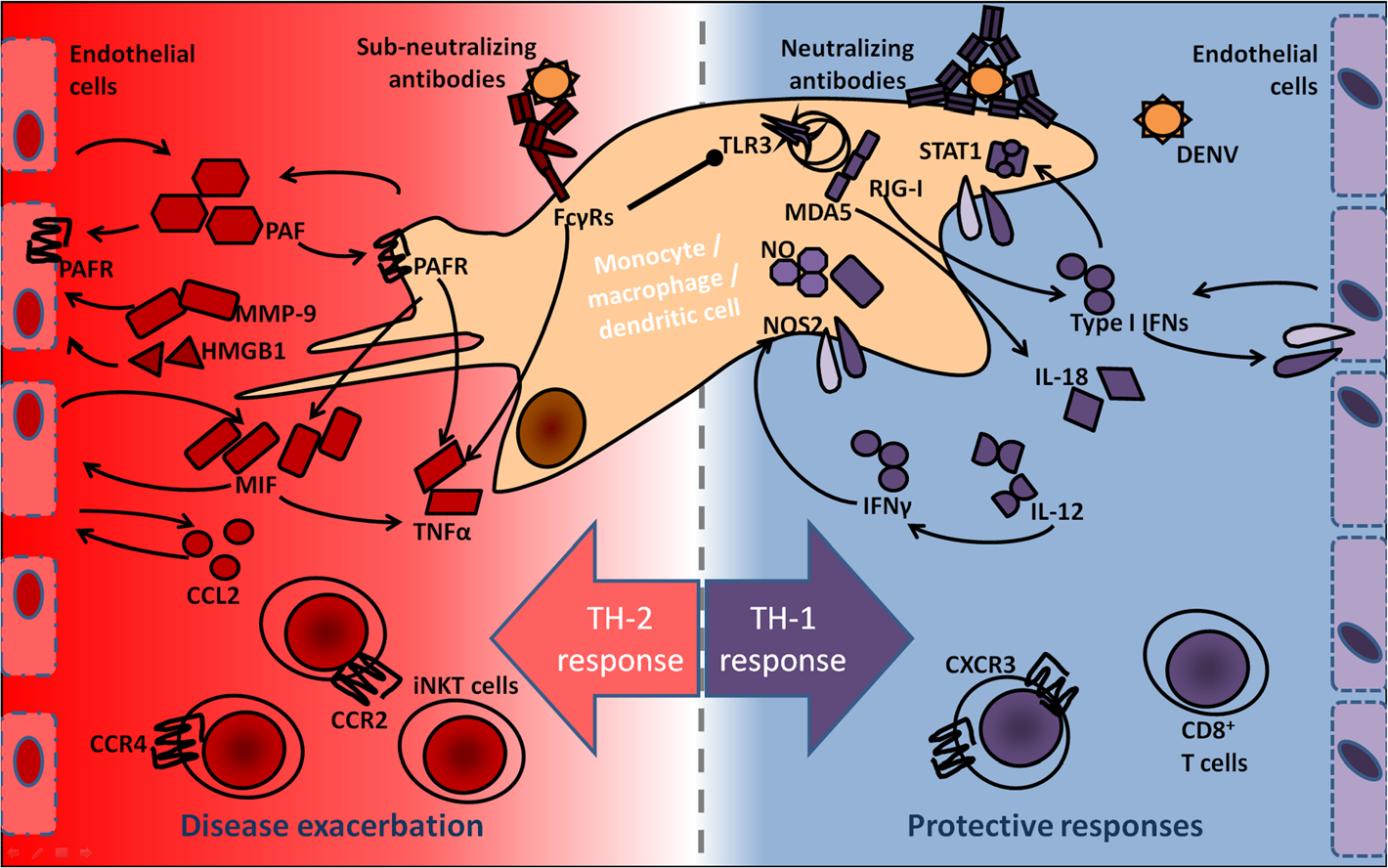
**Mechanisms in protective T**_**H**_ **1 responses and exacerbating T**_**H**_ **2 responses.**

Secondary dengue infections induce a T_H_2 type response mainly due to preexisting titers of semi-neutralizing I_g_G antibody [38,45,52]. Activated macrophages with up-regulated Fc-receptors engulf these complexes but become infected with the non-neutralized virus [36]. These infected macrophages secrete TNFα and T_H_2 type cytokines which suppress CTL and NK responses and lead to greatly increased vascular permeability, resulting in hemorrhage and shock (Figure 4) [52,65].

This cytokine storm follows a fever capable of restricting blood supply in synergy with TNFα to the low-perfusion, high-mutation rate clones with the most genetically unstable, and hence most dangerous characteristics [18,19]. In response, these low-perfusion clones may up-regulate the hypoxia-induced genes such as *Survivin,* thus, becoming more resistant to several therapeutic methods [20]. The DV-induced cytokine storm could enhance the effects of a variety of cancer immunotherapy approaches as follows: (1) direct targeting of the tumor cells, (2) targeting tumors in synergy with hyperthermia (TNFα), (3) expanding anti-tumor effector CTL, or (4) inducing tumor-reactive LAK or CIK cells, and (5) overcoming many of the immune evasion mechanisms employed by the tumor cells (Table 2) [43,66].

These immune evasion mechanisms are responsible for the lack of efficacy seen with most immunotherapy platforms [12,66]. As researchers come to understand the cancer therapy area at a deeper level, it is apparent that single-axis attacks play to cancer’s strengths of low perfusion and high mutation rates [17,20]. Therefore, a multi-pronged approach to cancer therapy is required to address all the immune evasion methods. The high levels of IFNα and IFNγ are capable of restoring Class I MHC levels essential for CTL recognition and lysis [67,68]. Additionally, using DV as an adjuvant to activate many pathways working in synergy could support the eradication of mutated tumor cells, improving the clinical efficacy of the cancer immunotherapy [56,69].

The ability of DV to attach to, and infect endothelial cells expressing Heparin-Sulfate Glycosaminoglycans (HSPG) may address a crucial impediment to clinical response to immunotherapy [57]. Effective immunotherapy requires both an activated CTL and successful trafficking of these CTL into tumor sites. To accomplish this, HSPG are expressed at higher levels in melanoma metastatic lesions, and by infecting tumor vessels, DV may interrupt perfusion and attract infiltrating DC and CTL to tumor areas. After DV is attracted to the tumor areas, infiltration then becomes critical, as a review study found that 93.8% of melanoma samples showed no P and E-Selectin expression on CD31^+^ vessels [70]. With Dengue triggering a stronger immune response through TNFα, P and E-Selectins are also upregulated, increasing attachment rates for CTL, to allow them to infiltrate the tumor areas. The tumor areas were also deficient in ICAM-1 expression. Induction of activated CTL that cannot traffic to or infiltrate tumor areas will lead to a sub-optimal response. By helping to up-regulate P and E selectins, as well as ICAM-1, Dengue may help address the critical barrier to successful immunotherapy [64].

Additional evidence of DV’s adjuvant potential can be found by analyzing gene expression profiles of cancer patients [25]. During active immunotherapy, researchers found that responding patients displayed a unique signature of immune-related genes. These include: Interferon-γ, interferon-regulatory factor-1 (IRF-1)/STAT-1 pathway, including expression of HLA Class I and II genes associated with effector function (TIAR, NK4, and granulysin) [71]. These genes represent the same pathways activated by acute allograft rejection. Interferon-regulatory factor-2 (IRF-2) family genes, on the other hand, are frequently up-regulated in non-responding lesions, and are associated with chronic inflammation [27].

Primary DV infections induce a T_H_1 type response with specific expression patterns favoring induction of potent anti-tumor immune responses (Table 3). Another factor to consider is the phenotype of the vaccine-induced effector CTL. Most immunotherapy strategies utilize self-antigens in a neutral or T_H_2 cytokine environment, and the vaccine-induced T cells often have a regulatory CD4^+^CD25^+^ Fox^p3+^ profile that can degrade, rather than enhance, the tumor immune response [72-74]. IL-12 appears to have a critical role in shifting DC polarity to T_H_1, and DV induces high levels of this cytokine [75]. For optimum DC therapy, large numbers of antigen-loaded DC must traffic to the white pulp region of the spleen [76-78]. A successful immunotherapy approach must be able to induce specific and non-specific effector cells, possessing the ability to overcome the immunosuppressive tumor microenvironment [13,60]. The effector cells induced during DV infection were found to express markers associated with effective anti-tumor immune responses (Table 4) [79].

**Table 3:** **Gene expression changes in DV infection**

**Gene**	**Symbol**	**Function**	**Fold-increase during DV Infection**	**Immunotherapy significance**
IL-1beta	IL-1β	Inflammation	9.80 [52]	Fever, Vascular permeability [35]
Interleukin-2	IL-2	T cell growth	3.97 [46]	CTL growth, NK Activation [80]
Interleukin-12	CLMF/CLMF2	Shift to T_H_1 Cytokines	2.20 [46]	T_H_1 cytokine storm [55]
Interferon γ	IFNG	Up-regulate Class I MHC	2.85 [46]	CTL recognize HLA + peptide [11]
CD8 antigen	CD8β1	CTL Co-receptor	5.74 [52]	Stabilizes MHC-TCR bond [68]
Inducible T cell co-stimulatory ligand	ICOSLG	Non-naïve T cell activation T_H_1 cytokines	2.44 [52]	Optimize response of CTL [25]
Chemokine ligand 3	CCL3	T-cell activation Autoimmunity	4.64 [52]	Marker of vaccine response [25]
Chemokine ligand 5	CCL5	T-cell localization	7.33 [52]	T-cell attraction to tumor area [81]
TRAIL	TRAIL	Apoptosis Induction	42.0 [65]	Induces Apoptosis in Tumor Cells [82]
Interferon-protein-10	IP10	T cell activation, Localization	46.0 [65]	Levels correlate w/clinical outcome in RCC [71]
Granulysin	GNLY	Apoptosis Induction	4.90 [52]	Induces Apoptosis [52]
Granzyme A	GZMA	Target cell lysis	4.61 [52]	Effector CTL [25]
MHC class II DRα	HLA-DRA	Antigen Presentation	7.26 [52]	Antigen Presentation [52]
MHC class II DPα1	HLA-DPα1	MHC peptide Display	4.58 [52]	Antigen (Ag) Presentation [52]
MHC class I DPβ1	HLA-DPβ1	MHC peptide Display	2.72 [52]	Antigen Presentation [52]
ζ chain kinase	ZAP70	TCR signal transduction	3.03 [52]	Restores CTL signaling [52]

**Table 4:** **Markers of activated lymphocytes in DV infection** ***in vivo*** [40]

**Marker**	**Control**	**Dengue fever**	**Fold-Change**	**Relevance**
CD8+ CD44^+^62 L^−^	6.5	24	3.7	Effector/memory set/Traffic to inflamed tissue
CD4+ CD44 + CD62L^LO^	7.1	22.4	3.2	Helper/Traffic to Inflamed Tissue
HLA-DR^+^ (CD8)	5.8	12.5	2.2	Activation Marker
Tia-1 (CD8)	6.6	20.3	3.1	Cytolytic effector
VLA-4 CD8)	42.7	61.0	1.4	CTL trafficking to inflamed sites
ICAM-1 (CD8)	18.8	29.8	1.6	Cell adhesion
LFA-1 (CD8)	52.9	71.4	1.4	Binds to ICAM-1 on target, co-stimulation

While properly activated effector cells of the adaptive and innate immune systems are powerful immunotherapy agents, they are subject to certain limitations. Activation-Induced Cell Death (AICD) of CTL involves up-regulation of *Bax* apoptosis-inducing gene, and down-regulation of *Bcl-2*, which counteracts *Bax* [58,83]. High levels of IL-2 pre-dispose CTL to AICD, and although Dengue Virus induces high levels of IL-15 to counteract this pathway, the majority of CTL induced during the proposed vaccine therapy will eventually die off due to AICD [58,80]. Another limitation is the requirements for adequate levels of HLA and cognate tumor antigen expression on tumor target cells [11]. DV induces high levels of HLA expression through IFNα and IFNγ [37,41].

### Dengue as an apoptosis-inducing agent

**Figure 5:**
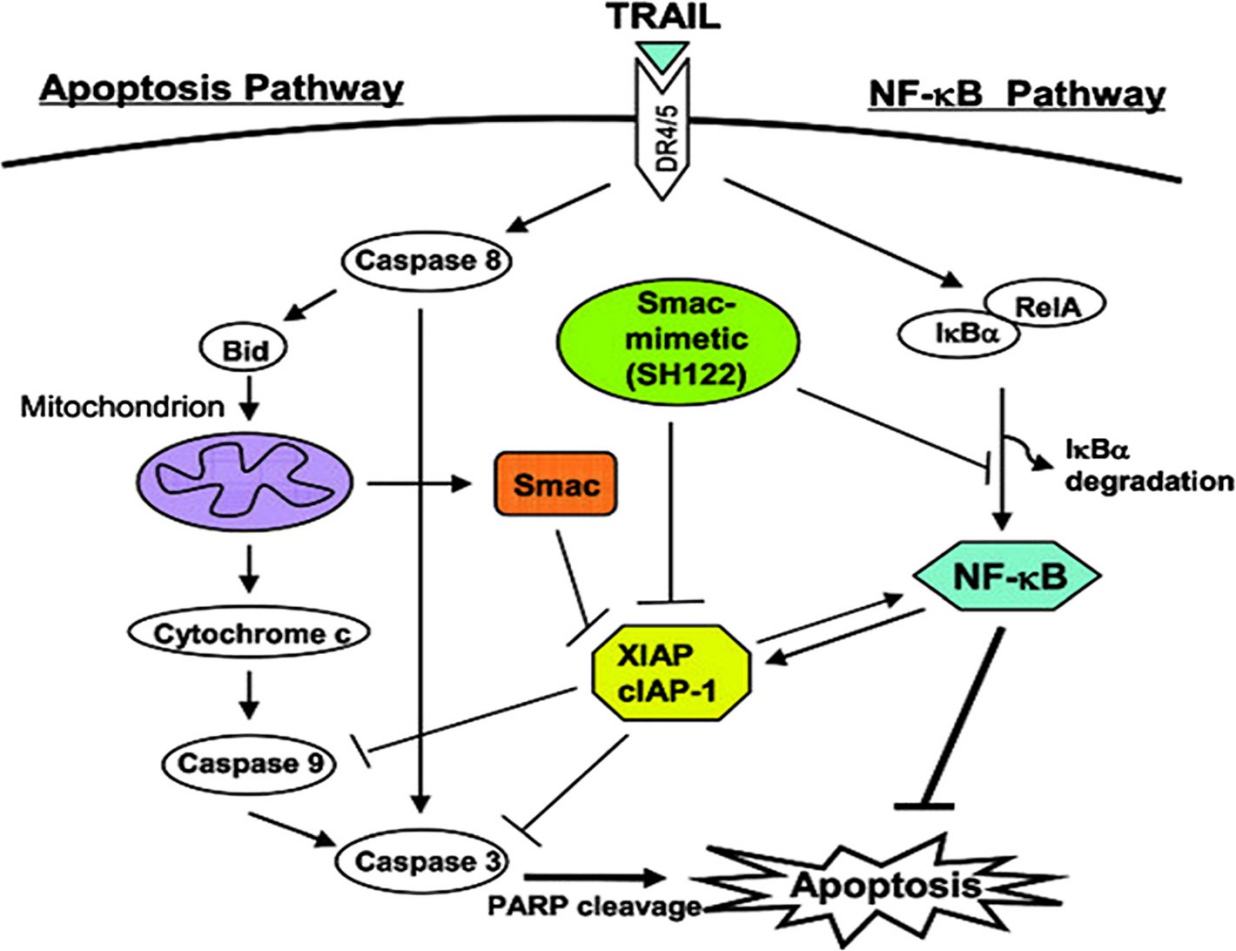
**Representation of Apo2L/TRAIL apoptotic pathway and its relation to the NF-κβpathway.** With permission from Springer Images for non-commercial use [86].

An ideal combination immunotherapy would combine physiological mechanisms with activated effectors of the adaptive and innate immune system, plus an apoptosis-inducing agent specifically targeted to tumor cells. TNF-related apoptosis-inducing ligand (TRAIL) is a member of the TNF superfamily of apoptosis-inducing agents, also including Fas (CD95. TNF [82] TRAIL/Apo2L binds to five TNF-family receptors on the cell surface: two of these, DR4 and DR5, are capable of transducing an apoptotic signal through the capsase-8 pathway when ligated with TRAIL [84]. The other three: decoy receptor-1 and 2, and the osteoprotegerin receptor, are incapable of inducing apoptotic signal and are thought to have a competitive inhibitory function [82,85] (Figure 5).

Dengue Virus induces high levels (300 pg/ml) of soluble TRAIL (sTRAIL) from a variety of cells including γδCTL, activated M_1_ macrophages and plasmacytoid DC (pDC) [41,87,88]. pDC are resistant to DV infection, but produce high levels of IFNα and membrane-bound TRAIL, giving them a killer or kpDC designation [41]. The non-classical γδ CTL, when activated by DV and West Nile Virus glycoproteins, secrete sTRAIL as their mechanism of action (MoA) against a wide variety of tumor cells [89,90]. While the precise amount of sTRAIL required to induce apoptosis has wide variation by tumor type, DR4/5 levels, and prior sensitization, the high levels of sTRAIL and factors to reduce resistance by Dengue may prove an effective mechanism of action to complement the vaccine therapy [83,91,92].

**Figure 6:**
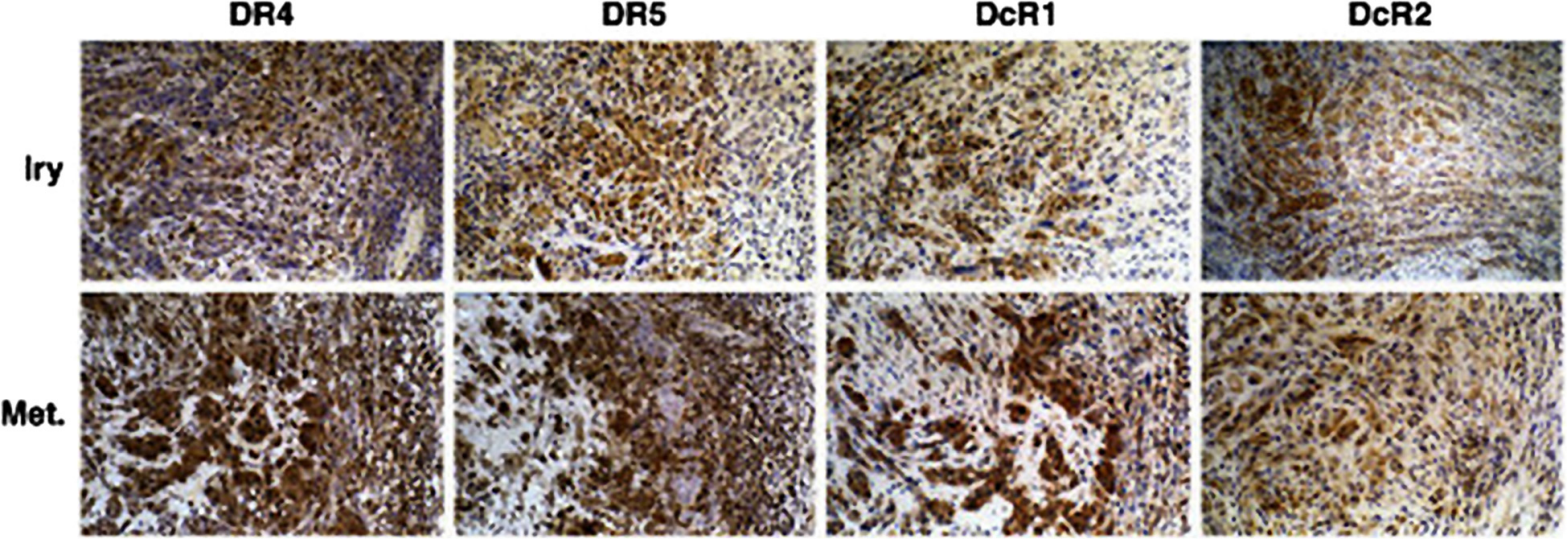
**H & E staining for Death Receptors DR4 and DR5, compared to Decoys DcR1 and DcR2.** In primary epithelial cells (top row), staining of DR4/5 is greater then DcR1/2. In metastatic lesions, (bottom row), the relative expressions are reversed, conferring resistance to TRAIL-mediated apoptosis. With permission from Springer Images for non-commercial use [93].

TRAIL has represented an attractive targeting approach, as it is capable of killing a wide range of tumor cells without harm to normal cells [84]. However, clinical trials with TRAIL have met with little success, as some cancer cells down-regulate DR4 and DR5 through activation of the NF-κβ pathway, allowing resistance to TRAIL [82]. Studies of melanoma lines showed an increase in decoy receptor and decrease in DR4/5 expression as lesions progressed [85]. For TRAIL to effectively induce apoptosis, tumor cells must be first sensitized by exposure to cytokines and other factors which up-regulate DR4/5 expression levels (Figure 6) [85].

DV activates multiple pathways known to help overcome resistance to TRAIL-mediated apoptosis of tumor cells. For example, DV replicates via a dsRNA intermediate, and this complex, while assembling in the Endoplasmic Reticulum, activates the TLR3 system [83,94]. Increased expression of TLR3 leads to suppression of DR4/5 inhibitory pathways and subsequent increases in TRAIL sensitivity [83]. In addition, DV envelope protein induces high levels of IL-27, a pleiotropic cytokine of the IL-6 family [95]. IL-27 acts to up-regulate TLR3 signaling on melanoma cells, and restores vulnerability to TRAIL-mediated apoptosis [83]. DV infection also stimulates the inducible Nitric Oxide (iNOS) pathway [91], which plays a pivotal role in remodeling vessel diameter and architecture during infection. iNOS is released by Macrophages and endothelial cells exposed to stimuli like Lipopolysaccharide binding protein (LPS) and IFNγ NO also sensitizes tumor cells to sTRAIL by inhibiting both NF-κΒ and the DR5 transcription repressor Yin Yang 1 [92]. This correlates with restoring sensitivity to TRAIL-mediated apoptosis by sTRAIL, or effector cells: γδCTL and kpDC, expressing membrane-bound TRAIL receptors [41,88,96].

DV also activates IFNβ, a multifunctional cytokine with a 10-fold higher affinity for the same receptor as IFNα [94]. IFNβ has similar antiviral properties in suppressing transcription of viral RNA, but is much more potent than IFNα in inducing apoptosis in tumor cells [97]. NO and IFNβ could act in a synergistic fashion during dengue infection. These molecules may work in tandem to overcome resistance to apoptosis mediated by the high levels of sTRAIL induced by M_1_ macrophages, pDC, and δγ CTL [82].

## Conclusions

In summary, the multiple DV-induced pathways overcoming the resistance to TRAIL-mediated apoptosis of tumor cells, combined with the high levels of TRAIL expressed on dengue effectors and sTRAIL released, could prove highly effective in eliminating tumor cells able to survive the multiple physiologic and cellular effector arms of the therapy. Thus, the approach employs not only multiple mechanisms of action, but entire systems: physiological, immunological, and apoptotic gene expression systems. A concentrated, coordinated effort to attack tumors on multiple pathways in a synergistic fashion could lead to higher response rates than current approaches attacking tumors along a single-axis pathway [98].

Primary DV infections allow a unique window of opportunity to employ both adaptive and innate immune responses directed against tumor cells, combined with physiological and death-receptor gene pathways. The fever and TNFα work in synergy to eliminate the most dangerous cells: those with high mutation rates and low perfusion; these tumor cells are the most resistant to systemic cancer therapies. TNFα also helps to remodel tumor microvasculature lacking the factors needed for CTL attachment and extravasation: P and E-Selectins, ICAM-1, VACM-1. Up-regulated Heparin Sulfate Glycosaminoglycan expression should increase virus infection in these areas. The high levels of T_H_1-type cytokines result in maximum stimulation of effector cells of both the innate and the adaptive immune systems. Simultaneously, these cytokines help to overcome the immune evasion and resistance mechanisms employed by the tumor cells. As a final mechanism of action, dengue virus induces high levels of sTRAIL, an apoptosis-inducing ligand binding to Death Receptor 4 (DR4) and Death Receptor 5 (DR5), expressed on tumor and virus- infected cells. While some tumor cells are resistant to sTRAIL, dengue also activates factors capable of overcoming this resistance to sTRAIL-mediated apoptosis.

A start-up biotechnology company (PrimeVax, LLC) has patented the use of dengue as a cancer immunotherapy agent in conjunction with autologous, tumor-lysate-pulsed dendritic cells. Dr. Duane Gubler, D.Sc., has provided PrimeVax LLC with a DEN-2 strain #1710 isolated during a Puerto Rican epidemic. This outbreak had 9,540 suspected cases of DV, with one suspected, but no confirmed deaths due to the virus [99]. PrimeVax is currently planning a Phase I trial in advanced melanoma, combining autologous tumor lysate-pulsed DC with DV as an adjuvant. The results from this trial may unlock some of the secrets of tumor immune evasion that have been obstacles to achieving better clinical responses.

Some future issues that need to be further explored include:

1. Although epidemiological and experimental data for DV infections appear to have an acceptable safety profile, only a clinical trial will provide the data required to properly evaluate the actual efficacy and safety of this novel approach.
2. It is possible that the cytokine release induced by the innate stimulator dengue virus will cause endothelial leakage similar to the side effect that has been seen in CAR-T trials and IL-2. However, the dengue cytokine storm is an intrinsic factor, and is therefore subject to internal regulation pathways such as increased levels of IL-4 and IL-10 during the recovery phase. Bolus IL-2 and CAR-T cells are *extrinsic* factors which are more difficult to regulate by internal feedback/inhibition loops. This difference may lead to a lower rate of serious effects related to endothelial leakage, especially with careful monitoring of BP, hematocrit, and fluid balance.
3. PrimeVax is preparing to file an IND with the FDA Center for Biologics Evaluation and Research (CBER) for a small Phase 1 trial using their approach on advanced melanoma patients progressing on current therapies.
4. If this approach has a favorable safety and efficacy profile, PrimeVax intends to expand their indication targets to include breast, prostate, and brain cancer, as well as other solid tumors and leukemias/lymphomas.
5. The principles of PrimeVax’s therapy can be applied to *any* type of solid tumor or leukemia/lymphoma by altering the antigens used to pulse the dendritic cells.
6. Whole tumor cell lysates can be utilized in order to present the full range of class-I and class II peptides to CTL, as well as to employ epitope spreading principles to target immune-escape variants.

## References

[1] Gubler DJ (1998). Dengue and dengue hemorrhagic fever. Clin Microbiol Rev.

[2] Wald TG, Shult P, Krause P, Miller BA, Drinka P, Gravenstein S (1995). A rhinovirus outbreak among residents of a long-term care facility. Ann Intern Med.

[3] Wolchok JD, Kluger H, Callahan MK, Postow MA, Rizvi NA, Lesokhin AM, Segal NH, Ariyan CE, Gordon RA, Reed K, Burke MM, Caldwell A, Kronenberg SA, Agunwamba BU, Zhang X, Lowy I, Inzunza HD, Feely W, Horak CE, Hong Q, Korman AJ, Wigginton JM, Gupta A, Sznol M (2013). Nivolumab plus ipilimumab in advanced melanoma. N Engl J Med.

[4] Corsello SM, Barnabei A, Marchetti P, De Vecchis L, Salvatori R, Torino F (2013). Endocrine side effects induced by immune checkpoint inhibitors. J Clin Endocrinol Metab.

[5] Fellner C (2012). Ipilimumab (yervoy) prolongs survival in advanced melanoma: serious side effects and a hefty price tag may limit its use. P T.

[6] Gardner TA, Elzey BD, Hahn NM (2012). Sipuleucel-T (Provenge) autologous vaccine approved for treatment of men with asymptomatic or minimally symptomatic castrate-resistant metastatic prostate cancer. Hum Vaccin Immunother.

[7] Patel SP, Woodman SE (2011). Profile of ipilimumab and its role in the treatment of metastatic melanoma. Drug Des Devel Ther.

[8] Liechtenstein T, Dufait I, Bricogne C, Lanna A, Pen J, Breckpot K, Escors D (2012). PD-L1/PD-1 Co-Stimulation, a Brake for T cell Activation and a T cell Differentiation Signal. J Clin Cell Immunol.

[9] Chmielewski M, Hombach AA, Abken H (2013). Antigen-Specific T-Cell Activation Independently of the MHC: Chimeric Antigen Receptor-Redirected T Cells. Front Immunol.

[10] Aris M, Barrio MM, Mordoh J (2012). Lessons from cancer immunoediting in cutaneous melanoma. Clin Dev Immunol.

[11] Algarra I, Garcia-Lora A, Cabrera T, Ruiz-Cabello F, Garrido F (2004). The selection of tumor variants with altered expression of classical and nonclassical MHC class I molecules: implications for tumor immune escape. Cancer Immunol Immunother.

[12] Ellem KA, Schmidt CW, Li CL, Misko I, Kelso A, Sing G, Macdonald G, O’Rourke MG (1998). The labyrinthine ways of cancer immunotherapy–T cell, tumor cell encounter: “how do I lose thee? Let me count the ways”. Adv Cancer Res.

[13] Iancu EM, Baumgaertner P, Wieckowski S, Speiser DE, Rufer N (2011). Profile of a serial killer: cellular and molecular approaches to study individual cytotoxic T-cells following therapeutic vaccination. J Biomed Biotechnol.

[14] Jessy T (2011). Immunity over inability: The spontaneous regression of cancer. J Nat Sci Biol Med.

[15] McCarthy EF (2006). The toxins of William B. Coley and the treatment of bone and soft-tissue sarcomas. Iowa Orthop J.

[16] Bai XF, Bender J, Liu J, Zhang H, Wang Y, Li O, Du P, Zheng P, Liu Y (2001). Local costimulation reinvigorates tumor-specific cytolytic T lymphocytes for experimental therapy in mice with large tumor burdens. J Immunol.

[17] Durand RE (2001). Intermittent blood flow in solid tumours–an under-appreciated source of ‘drug resistance’. Cancer Metastasis Rev.

[18] Vaupel P, Kallinowski F (1987). Physiological effects of hyperthermia. Recent Results Cancer Res.

[19] Dudar TE, Jain RK (1984). Differential response of normal and tumor microcirculation to hyperthermia. Cancer Res.

[20] Chan N, Bristow RG (2010). “Contextual” synthetic lethality and/or loss of heterozygosity: tumor hypoxia and modification of DNA repair. Clin Cancer Res.

[21] Watanabe N, Niitsu Y, Umeno H, Kuriyama H, Neda H, Yamauchi N, Maeda M, Urushizaki I (1988). Toxic effect of tumor necrosis factor on tumor vasculature in mice. Cancer Res.

[22] Hober D, Delannoy AS, Benyoucef S, De Groote D, Wattre P (1996). High levels of sTNFR p75 and TNF alpha in dengue-infected patients. Microbiol Immunol.

[23] Ganss R, Ryschich E, Klar E, Arnold B, Hammerling GJ (2002). Combination of T-cell therapy and trigger of inflammation induces remodeling of the vasculature and tumor eradication. Cancer Res.

[24] Simmons CP, Farrar JJ, Nguyen VV, Wills B (2012). Dengue. N Engl J Med.

[25] Bedognetti D, Wang E, Sertoli MR, Marincola FM (2010). Gene-expression profiling in vaccine therapy and immunotherapy for cancer. Expert Rev Vaccines.

[26] Niakan B (1998). A mechanism of the spontaneous remission and regression of cancer. Cancer Biother Radiopharm.

[27] Clancy T, Pedicini M, Castiglione F, Santoni D, Nygaard V, Lavelle TJ, Benson M, Hovig E (2011). Immunological network signatures of cancer progression and survival. BMC Med Genomics.

[28] Butz EA, Bevan MJ (1998). Massive expansion of antigen-specific CD8+ T cells during an acute virus infection. Immunity.

[29] Kaufman HL, Bines SD (2010). OPTIM trial: a Phase III trial of an oncolytic herpes virus encoding GM-CSF for unresectable stage III or IV melanoma. Future Oncol.

[30] Prestwich RJ, Errington F, Diaz RM, Pandha HS, Harrington KJ, Melcher AA, Vile RG (2009). The case of oncolytic viruses versus the immune system: waiting on the judgment of Solomon. Hum Gene Ther.

[31] Wong HH, Lemoine NR, Wang Y (2010). Oncolytic Viruses for Cancer Therapy: Overcoming the Obstacles. Viruses.

[32] Kim DW, Krishnamurthy V, Bines SD, Kaufman HL (2010). TroVax, a recombinant modified vaccinia Ankara virus encoding 5 T4: lessons learned and future development. Hum Vaccin.

[33] Leitmeyer KC, Vaughn DW, Watts DM, Salas R, Villalobos I, De C, Ramos C, Rico-Hesse R (1999). Dengue virus structural differences that correlate with pathogenesis. J Virol.

[34] Manson A (2014). Manson’s Tropical Diseases.

[35] Chang DM, Shaio MF (1994). Production of interleukin-1 (IL-1) and IL-1 inhibitor by human monocytes exposed to dengue virus. J Infect Dis.

[36] Chen YC, Wang SY (2002). Activation of terminally differentiated human monocytes/macrophages by dengue virus: productive infection, hierarchical production of innate cytokines and chemokines, and the synergistic effect of lipopolysaccharide. J Virol.

[37] Kurane I, Innis BL, Nimmannitya S, Nisalak A, Meager A, Janus J, Ennis FA (1991). Activation of T lymphocytes in dengue virus infections. High levels of soluble interleukin 2 receptor, soluble CD4, soluble CD8, interleukin 2, and interferon-gamma in sera of children with dengue. J Clin Invest.

[38] Dalrymple NA, Mackow ER (2012). Endothelial cells elicit immune-enhancing responses to dengue virus infection. J Virol.

[39] Pacsa AS, Agarwal R, Elbishbishi EA, Chaturvedi UC, Nagar R, Mustafa AS (2000). Role of interleukin-12 in patients with dengue hemorrhagic fever. FEMS Immunol Med Microbiol.

[40] Azeredo EL, De Oliveira-Pinto LM, Zagne SM, Cerqueira DI, Nogueira RM, Kubelka CF (2006). NK cells, displaying early activation, cytotoxicity and adhesion molecules, are associated with mild dengue disease. Clin Exp Immunol.

[41] Gandini M, Gras C, Azeredo EL, Pinto LM, Smith N, Despres P, da Cunha RV, de Souza LJ, Kubelka CF, Herbeuval JP (2013). Dengue Virus activates membrane TRAIL localizatoin and IFN-alpha production by human plasmacytoid dendritic cells in vitro and in vivo. PLoS Negl Trop Dis.

[42] Kurane I, Janus J, Ennis FA (1992). Dengue virus infection of human skin fibroblasts in vitro production of IFN-beta, IL-6 and GM-CSF. Arch Virol.

[43] Copier J, Bodman-Smith M, Dalgleish A (2011). Current status and future applications of cellular therapies for cancer. Immunotherapy.

[44] Rosenberg SA, Yang JC, Restifo NP (2004). Cancer immunotherapy: moving beyond current vaccines. Nat Med.

[45] Bozza FA, Cruz OG, Zagne SM, Azeredo EL, Nogueira RM, Assis EF, Bozza PT, Kubelka CF (2008). Multiplex cytokine profile from dengue patients: MIP-1beta and IFN-gamma as predictive factors for severity. BMC Infect Dis.

[46] Chen J, Ng MM, Chu JJ (2008). Molecular profiling of T-helper immune genes during dengue virus infection. Virol J.

[47] Wills BA, Nguyen MD, Ha TL, Dong TH, Tran TN, Le TT, Tran VD, Nguyen TH, Nguyen VC, Stepniewska K, White NJ, Farrar JJ (2005). Comparison of three fluid solutions for resuscitation in dengue shock syndrome. N Engl J Med.

[48] da Rosa AP T, Vasconcelos PF, Travassos Da Rosa ES, Rodrigues SG, Mondet B, Cruz AC, Sousa MR, Travassos Da Rosa JF (2000). Dengue epidemic in Belem, Para, Brazil, 1996-97. Emerg Infect Dis.

[49] Lee IK, Liu JW, Yang KD (2009). Clinical characteristics, risk factors, and outcomes in adults experiencing dengue hemorrhagic fever complicated with acute renal failure. Am J Trop Med Hyg.

[50] Wang CC, Liu SF, Liao SC, Lee IK, Liu JW, Lin AS, Wu CC, Chung YH, Lin MC (2007). Acute respiratory failure in adult patients with dengue virus infection. Am J Trop Med Hyg.

[51] Overwijk WW, Theoret MR, Finkelstein SE, Surman DR, de Jong LA, Vyth-Dreese FA, Dellemijn TA, Antony PA, Spiess PJ, Palmer DC, Heimann DM, Klebanoff CA, Yu Z, Hwang LN, Feigenbaum L, Kruisbeek AM, Rosenberg SA, Restifo NP (2003). Tumor regression and autoimmunity after reversal of a functionally tolerant state of self-reactive CD8+ T cells. J Exp Med.

[52] Ubol S, Masrinoul P, Chaijaruwanich J, Kalayanarooj S, Charoensirisuthikul T, Kasisith J (2008). Differences in global gene expression in peripheral blood mononuclear cells indicate a significant role of the innate responses in progression of dengue fever but not dengue hemorrhagic fever. J Infect Dis.

[53] Librarty D (2001). Human dendritic cells are activated by Dengue Virus infection: enhancement by Gamma Interferon and implications for disease pathogenesis. J Virology.

[54] Green S, Pichyangkul S, Vaughn DW, Kalayanarooj S, Nimmannitya S, Nisalak A, Kurane I, Rothman AL, Ennis FA (1999). Early CD69 expression on peripheral blood lymphocytes from children with dengue hemorrhagic fever. J Infect Dis.

[55] Wagner SN, Schultewolter T, Wagner C, Briedigkeit L, Becker JC, Kwasnicka HM, Goos M (1998). Immune response against human primary malignant melanoma: a distinct cytokine mRNA profile associated with spontaneous regression. Lab Invest.

[56] Sinkovics JG, Horvath JC (2005). Human natural killer cells: a comprehensive review. Int J Oncol.

[57] Kelley JF, Kaufusi PH, Nerurkar VR (2012). Dengue hemorrhagic fever-associated immunomediators induced via maturation of dengue virus nonstructural 4B protein in monocytes modulate endothelial cell adhesion molecules and human microvascular endothelial cells permeability. Virology.

[58] Kovalovich K, Li W, DeAngelis R, Greenbaum LE, Ciliberto G, Taub R (2001). Interleukin-6 protects against Fas-mediated death by establishing a critical level of anti-apoptotic hepatic proteins FLIP, Bcl-2, and Bcl-xL. J Biol Chem.

[59] von Russum A, Krall R, Escalante NK, Choy JC (2011). Inflammatory cytokines determine the susceptibility of human CD8 T cells to Fas-mediated activation-induced cell death through modulation of FasL and c-FLIP(S) expression. J Biol Chem.

[60] West EJ, Scott KJ, Jennings VA, Melcher AA (2011). Immune activation by combination human lymphokine-activated killer and dendritic cell therapy. Br J Cancer.

[61] Lindau D (2013). The Immunosuppressive tumor network: Myeloid-Derived Suppressor Cells, regulatory T cells and Natural Killer Cells. J Immunol.

[62] Suwannasaen D, Romphruk A, Leelayuwat C, Lertmemongkolchai G (2010). Bystander T cells in human immune responses to dengue antigens. BMC Immunol.

[63] Medema JP, de Jong J, Peltenburg LT, Verdegaal EM, Gorter A, Bres SA, Franken KL, Hahne M, Albar JP, Melief CJ, Offringa R (2001). Blockade of the granzyme B/perforin pathway through overexpression of the serine protease inhibitor PI-9/SPI-6 constitutes a mechanism for immune escape by tumors. Proc Natl Acad Sci U S A.

[64] Hamai A, Meslin F, Benlalam H, Jalil A, Mehrpour M, Faure F, Lecluse Y, Vielh P, Avril MF, Robert C, Chouaib S (2008). ICAM-1 has a critical role in the regulation of metastatic melanoma tumor susceptibility to CTL lysis by interfering with PI3K/AKT pathway. Cancer Res.

[65] Becerra A, Warke RV, Martin K, Xhaja K, de Bosch N, Rothman AL, Bosch I (2009). Gene expression profiling of dengue infected human primary cells identifies secreted mediators in vivo. J Med Virol.

[66] Turcotte S, Rosenberg SA (2011). Immunotherapy for metastatic solid cancers. Adv Surg.

[67] Becquart P, Wauquier N, Nkoghe D, Ndjoyi-Mbiguino A, Padilla C, Souris M, Leroy EM (2010). Acute dengue virus 2 infection in Gabonese patients is associated with an early innate immune response, including strong interferon alpha production. BMC Infect Dis.

[68] Yong X, Xiao YF, Luo G, He B, Lu MH, Hu CJ, Guo H, Yang SM (2012). Strategies for enhancing vaccine-induced CTL antitumor immune responses. J Biomed Biotechnol.

[69] Mesiano G, Todorovic M, Gammaitoni L, Leuci V, Giraudo Diego L, Carnevale-Schianca F, Fagioli F, Piacibello W, Aglietta M, Sangiolo D (2012). Cytokine-induced killer (CIK) cells as feasible and effective adoptive immunotherapy for the treatment of solid tumors. Expert Opin Biol Ther.

[70] Weishaupt C, Munoz KN, Buzney E, Kupper TS, Fuhlbrigge RC (2007). T-cell distribution and adhesion receptor expression in metastatic melanoma. Clin Cancer Res.

[71] Wolf B, Schwarzer A, Cote AL, Hampton TH, Schwaab T, Huarte E, Tomlinson CR, Gui J, Fisher JL, Fadul CE, Hamilton JW, Ernstoff MS (2012). Gene expression profile of peripheral blood lymphocytes from renal cell carcinoma patients treated with IL-2, interferon-alpha and dendritic cell vaccine. PLoS One.

[72] Disis ML (2011). Immunologic biomarkers as correlates of clinical response to cancer immunotherapy. Cancer Immunol Immunother.

[73] Chakraborty NG, Li L, Sporn JR, Kurtzman SH, Ergin MT, Mukherji B (1999). Emergence of regulatory CD4+ T cell response to repetitive stimulation with antigen-presenting cells in vitro: implications in designing antigen-presenting cell-based tumor vaccines. J Immunol.

[74] Steinbrink K, Jonuleit H, Muller G, Schuler G, Knop J, Enk AH (1999). Interleukin-10-treated human dendritic cells induce a melanoma-antigen-specific anergy in CD8(+) T cells resulting in a failure to lyse tumor cells. Blood.

[75] Pasca A (2012). Role of Interleukin-12 in patients with Dengue Hemorrhagic Fever. FEMS Immunol Med Microbiol.

[76] Creusot RJ, Yaghoubi SS, Chang P, Chia J, Contag CH, Gambhir SS, Fathman CG (2009). Lymphoid-tissue-specific homing of bone-marrow-derived dendritic cells. Blood.

[77] Morse MA, Coleman RE, Akabani G, Niehaus N, Coleman D, Lyerly HK (1999). Migration of human dendritic cells after injection in patients with metastatic malignancies. Cancer Res.

[78] Verdijk P, Aarntzen EH, Lesterhuis WJ, Boullart AC, Kok E, van Rossum MM, Strijk S, Eijckeler F, Bonenkamp JJ, Jacobs JF, Blokx W, Vankrieken JH, Joosten I, Boerman OC, Oyen WJ, Adema G, Punt CJ, Figdor CG, de Vries IJ (2009). Limited amounts of dendritic cells migrate into the T-cell area of lymph nodes but have high immune activating potential in melanoma patients. Clin Cancer Res.

[79] Azeredo EL, Zagne SM, Alvarenga AR, Nogueira RM, Kubelka CF, de Oliveira-Pinto LM (2006). Activated peripheral lymphocytes with increased expression of cell adhesion molecules and cytotoxic markers are associated with dengue fever disease. Mem Inst Oswaldo Cruz.

[80] Waldmann T (2002). The contrasting roles of IL-2 and IL-15 in the life and death of lymphocytes: implications for the immunotherapy of rheumatological diseases. Arthritis Res.

[81] Lapteva N, Huang XF (2010). CCL5 as an adjuvant for cancer immunotherapy. Expert Opin Biol Ther.

[82] Mellier G, Pervaiz S (2012). The three Rs along the TRAIL: resistance, re-sensitization and reactive oxygen species (ROS). Free Radic Res.

[83] Chiba Y, Mizoguchi I, Mitobe K, Higuchi K, Nagai H, Nishigori C, Mizuguchi J, Yoshimoto T (2013). IL-27 enhances the expression of TRAIL and TLR3 in human melanomas and inhibits their tumor growth in cooperation with a TLR3 agonist poly(I:C) partly in a TRAIL-dependent manner. PLoS One.

[84] Palacios C, Yerbes R, Sanchez-Perez T, Martin-Perez R, Cano-Gonzalez A, Lopez-Rivas A (2014). The long and winding road to cancer treatment: the trail system. Curr Pharm Des.

[85] Zhuang L, Lee CS, Scolyer RA, McCarthy SW, Zhang XD, Thompson JF, Screaton G, Hersey P (2006). Progression in melanoma is associated with decreased expression of death receptors for tumor necrosis factor-related apoptosis-inducing ligand. Hum Pathol.

[86] Dai Y (2009). A Smac-mimetic sensitizes prostate cancer cells to TRAIL-induced apoptosis via modulating both IAPs and NF-KappaB. BMC Cancer.

[87] Wong KL, Chen W, Balakrishnan T, Toh YX, Fink K, Wong SC (2012). Susceptibility and response of human blood monocyte subsets to primary dengue virus infection. PLoS One.

[88] Dokouhaki P, Schuh NW, Joe B, Allen CA, Der SD, Tsao MS, Zhang L (2013). NKG2D regulates production of soluble TRAIL by ex vivo expanded human gammadelta T cells. Eur J Immunol.

[89] Correia DV, Fogli M, Hudspeth K, da Silva MG, Mavilio D, Silva-Santos B (2011). Differentiation of human peripheral blood Vdelta1+ T cells expressing the natural cytotoxicity receptor NKp30 for recognition of lymphoid leukemia cells. Blood.

[90] Wang T, Welte T (2013). Role of natural killer and Gamma-delta T cells in West Nile virus infection. Viruses.

[91] Neves-Souza PC, Azeredo EL, Zagne SM, Valls-de-Souza R, Reis SR, Cerqueira DI, Nogueira RM, Kubelka CF (2005). Inducible nitric oxide synthase (iNOS) expression in monocytes during acute Dengue Fever in patients and during in vitro infection. BMC Infect Dis.

[92] Huerta-Yepez S, Vega M, Escoto-Chavez SE, Murdock B, Sakai T, Baritaki S, Bonavida B (2009). Nitric oxide sensitizes tumor cells to TRAIL-induced apoptosis via inhibition of the DR5 transcription repressor Yin Yang 1. Nitric Oxide.

[93] Vignesweran N (2007). Repression of tumor necrosis factor-related apoptosis-inducing ligand (TRAIL) but not its receptors during oral cancer progression. BMC Cancer.

[94] Nasirudeen AM, Wong HH, Thien P, Xu S, Lam KP, Liu DX (2011). RIG-I, MDA5 and TLR3 synergistically play an important role in restriction of dengue virus infection. PLoS Negl Trop Dis.

[95] Leng CH, Chen HW, Chang LS, Liu HH, Liu HY, Sher YP, Chang YW, Lien SP, Huang TY, Chen MY, Chou AH, Chong P, Liu SJ (2010). A recombinant lipoprotein containing an unsaturated fatty acid activates NF-kappaB through the TLR2 signaling pathway and induces a differential gene profile from a synthetic lipopeptide. Mol Immunol.

[96] Quast SA, Berger A, Buttstadt N, Friebel K, Schonherr R, Eberle J (2012). General Sensitization of melanoma cells for TRAIL-induced apoptosis by the potassium channel inhibitor TRAM-34 depends on release of SMAC. PLoS One.

[97] Caraglia M, Marra M, Tagliaferri P, Lamberts SW, Zappavigna S, Misso G, Cavagnini F, Facchini G, Abbruzzese A, Hofland LJ, Vitale G (2009). Emerging strategies to strengthen the anti-tumour activity of type I interferons: overcoming survival pathways. Curr Cancer Drug Targets.

[98] Tiwari AK, Roy HK (2012). Progress against cancer (1971-2011): how far have we come?. J Intern Med.

[99] Rigau-Perez JG, Clark GG (1992). Dengue activity in Puerto Rico, 1990. P R Health Sci J.

